# Referral decisions and its predictors related to orthopaedic care.  A retrospective study in a novel primary care setting

**DOI:** 10.1371/journal.pone.0227863

**Published:** 2020-01-23

**Authors:** Esther H. A. van den Bogaart, Marieke D. Spreeuwenberg, Mariëlle E. A. L. Kroese, Mark W. van den Boogaart, Tim A. E. J. Boymans, Dirk Ruwaard

**Affiliations:** 1 Department of Health Services Research, Care and Public Health Research Institute (CAPHRI), Faculty of Health Medicine and Life Sciences, Maastricht University, Maastricht, The Netherlands; 2 Research Centre for Technology in Care, Zuyd University of Applied Sciences, Heerlen, The Netherlands; 3 Department of Orthopaedic Surgery, Maastricht University Medical Centre+, Maastricht, The Netherlands; College of Medicine, University of Nigeria, NIGERIA

## Abstract

Due to the ageing population, the prevalence of musculoskeletal disorders will continue to rise, as well as healthcare expenditure. To overcome these increasing expenditures, integration of orthopaedic care should be stimulated. The Primary Care Plus (PC+) intervention aimed to achieve this by facilitating collaboration between primary care and the hospital, in which specialised medical care is shifted to a primary care setting. The present study aims to evaluate the referral decision following orthopaedic care in PC+ and in particular to evaluate the influence of diagnostic tests on this decision. Therefore, retrospective monitoring data of patients visiting PC+ for orthopaedic care was used. Data was divided into two periods; P1 and P2. During P2, specialists in PC+ were able to request additional diagnostic tests (such as ultrasounds and MRIs). A total of 2,438 patients visiting PC+ for orthopaedic care were included in the analysis. The primary outcome was the referral decision following PC+ (back to the general practitioner (GP) or referral to outpatient hospital care). Independent variables were consultation- and patient-related predictors. To describe variations in the referral decision, logistic regression modelling was used. Results show that during P2, significantly more patients were referred back to their GP. Moreover, the multivariable analysis show a significant effect of patient age on the referral decision (OR 0.86, 95% CI = 0.81–0.91) and a significant interaction was found between the treating specialist and the period (*p* = 0.015) and between patient’s diagnosis and the period (*p* ≤ 0.001). Despite the significant impact of the possibility of requesting additional diagnostic tests in PC+, it is important to discuss the extent to which the availability of diagnostic tests fits within the vision of PC+. In addition, selecting appropriate profiles for specialists and patients for PC+ are necessary to further optimise the effectiveness and cost of care.

## Introduction

Problems related to the musculoskeletal system are the second most common causes of disability and affect more than 1.7 billion people worldwide [1]. Most musculoskeletal disorders are associated with severe long-term pain and physical disability that affects an individual’s daily life [2–4] and are a major cause of work disability and absence, which leads to loss of productivity [2, 3, 5, 6]. Due to the ageing population in developed countries, the prevalence of musculoskeletal disorders will rise [7]. This increasing prevalence will lead to higher demand for health care services since individuals with musculoskeletal problems are among the highest users of care [8] and to a rise in healthcare costs [9].

In the Netherlands, musculoskeletal problems are a significant factor in high healthcare costs [10], with 1.1 billion euros spent on osteoarthritis in 2011, corresponding to 1.2% of total healthcare costs [11]. Many general practice consultations are therefore related to problems of the musculoskeletal system [12, 13]. Because Dutch general practitioners (GPs) have a gatekeeping role [14], one of the challenges facing them is deciding when to refer patients with musculoskeletal problems and to which medical speciality [15]. A study by Roland et al. [16] showed that, according to medical specialists, almost 25% of GP referrals to orthopaedics were unnecessary and primary care management was more appropriate. GP competency also varies significantly with respect to diagnosing and treating musculoskeletal disorders [17]. Due to rapid developments in diagnostics and treatment, it is unrealistic for GPs to be constantly informed about all possibilities [18]. These knowledge gaps that arise in primary care may lead to referrals to outpatient hospital care for diagnosis and/or treatment that GPs could actually provide if they had the right resources, training, and support [19].

To overcome this gap and to keep patients out of the hospital, the Dutch pioneer site “Blue Care” implemented Primary Care Plus (PC+) to share and embed specialist knowledge into primary care [20, 21]. PC+ involves hospital specialists providing consultations in the primary care setting, with a minimum of diagnostic tools, to prevent unnecessary referrals to outpatient hospital care. With this, outpatient hospital care is shifted to a more accessible primary care setting [22, 23].

Considering the novelty of PC+, this study examines whether orthopaedic care is suitable to be shifted to the primary care setting. Therefore, this study aims to evaluate the referral decision following orthopaedic care in PC+ in order to determine to what extent patients are referred back to their GP or how often a referral to hospital care is still necessary. In particular, this study will focus on the influence of the availability of diagnostic tests in PC+ on this referral decision, since contradictory results have been found in literature when it comes to the effect of a lack of diagnostic tools in orthopaedic care [21, 24]. Furthermore, other predictors, like consultation- and patient-related predictors of this decision will be studied as well. With these insights, PC+ for orthopaedic care can be further optimised.

## Materials and methods

### Design

The present retrospective study makes use of data on referral decisions during the period January 2015 to December 2017. The data were divided into two periods, P1 (from January 2015 to December 2016) and P2 (from January 2017 to December 2017). This distinction was based on the introduction of the possibility of orthopaedic surgeons, working in PC+, requesting additional diagnostic tests (such as ultrasounds and MRIs).

### Setting

In pioneer site Blue Care in the Maastricht-Heuvelland region, one of nine pioneer sites in the Netherlands, the primary care organisation Care in Development (in Dutch “*Zorg in Ontwikkeling*”), the Maastricht University Medical Centre+ (Maastricht UMC+), the health insurance company VGZ, and the patient representative foundation House of Care (in Dutch “*Huis voor de Zorg*”) work together. The Maastricht-Heuvelland region consists of 81 GPs working in 55 different GP practices caring for a population of about 170,000 people [25].

The Dutch healthcare system is characterised by the gatekeeping principle, meaning that a referral from the GP is required for hospital and specialist care, with the exception of emergency care [26]. Primary care, including GP consultation, is freely accessible for patients [27].

The region Maastricht-Heuvelland developed the PC+ intervention to substitute primary care for outpatient hospital care. The concept of PC+ started with a pilot in which four medical specialties performed consultations within GP practices [21, 28]. Orthopaedic care was one of the four specialties involved. Although the results of the feasibility study by van Hoof et al. [28] showed that PC+ seemed to be a promising intervention, problems of inefficiency and competitive restraints were found. Therefore, two independent PC+ centres located in the city of Maastricht were established in 2014. These two centres are located outside the hospital site, and the PC+ concept is quite similar to the well-known concept of specialist outreach clinics [29]. With the arrival of the PC+ centres, GPs within the region were able to refer patients to a medical specialist in a neutral primary care setting. The focus of this study was on orthopaedic care in the current PC+ setting.

### Intervention

**Fig 1:**
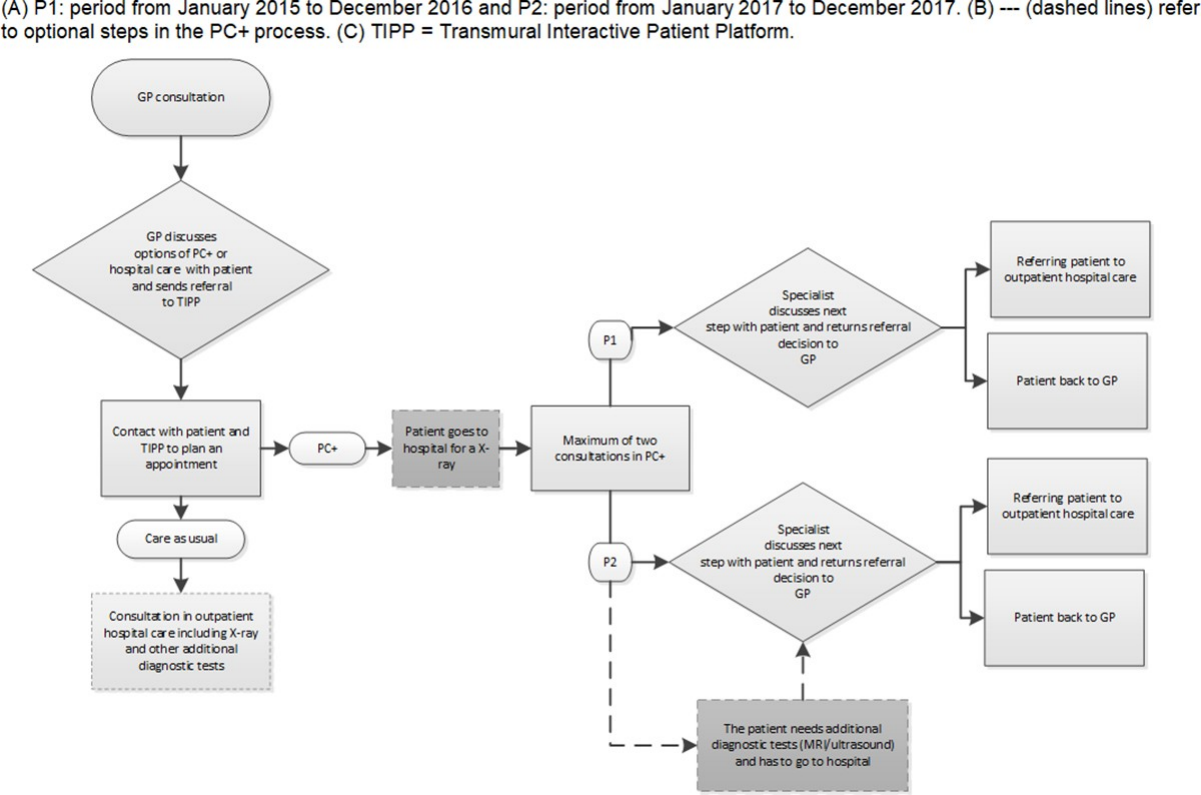
Flowchart of the PC+ referral process.

Fig 1 shows the total PC+ process, starting from referral to PC+ to the referral decision made by the specialist in PC+ during P1 and P2. In both periods, the decision to refer to PC+ was based on GP consultation with the patient. The referral was first sent to the Transmural Interactive Patient Platform (TIPP), which accordingly planned and registered referrals to medical specialists (either in PC+ or outpatient hospital care). When patients were referred to PC+, they needed to have a recent X-ray (not older than six months) of the affected body part; if patients did not have a recent X-ray, they first went to the hospital to get one. In PC+, patients were seen by an orthopaedic surgeon or a senior resident in orthopaedic surgery of Maastricht UMC+ for a maximum of two consultations. In the PC+ centre, care is claimed as primary care performance so consultations are not subjected to the patient’s deductible. Specialists treated patients and/or provided advice for GPs on further treatment strategies, and the GP retained responsibility for the patient.

If additional diagnostic tests were needed during P1, the referral decision following PC+ was automatic referral of the patient to outpatient hospital care. During P2, the specialist could request additional diagnostic tests within the PC+ setting. Patients had to visit the hospital only for these additional tests, and the test results determined whether a telephone consultation or follow-up consultation in PC+ was sufficient or whether a referral to outpatient hospital care for further diagnosis and/or treatment was necessary. An overview of the similarities and differences of P1 and P2 are shown in Table 1.

**Table 1:** Requirements, possibilities and consequences of diagnostics in PC+ during P1 and P2.

	Period 1	Period 2
	January 2015–December 2016	January 2017–December 2017
**Requirements for a consultation in PC+**	Patients need to have a recent X-ray (not older than six months) of the affected body part.	Patients need to have a recent X-ray (not older than six months) of the affected body part.
**Additional diagnostic possibilities**	No (or limited) possibility to request additional diagnostic tests (MRI & ultrasound).	Possibility to request additional diagnostic tests (MRI & ultrasound).
**Consequences if additional diagnostics are needed**	If additional diagnostics are required, patients are referred to outpatient hospital care.	If additional diagnostics are required, patients are referred to outpatient hospital care for diagnostic purposes only. Follow-up consultations can take place at PC+.

### Data collection

In this study, the data of all patients visiting PC+ for orthopaedic care were collected. The data of the two independent PC+ centres were merged into one data set. These data consist of patient age, gender, final established diagnosis, number of consultations in PC+, treating specialist, and the referral decision following PC+. The patient diagnosis was labelled according to the diagnosis group classification list, which is part of the Diagnosis Treatment Combination (DBC) [30].

### Outcome measures

The primary outcome of the present study was the referral decision following PC+ (back to the GP or referral to outpatient hospital care). Independent variables (or predictors) were consultation-related factors (number of consultations in PC+ and treating specialist) and patient-related factors (patient age, gender, and final established diagnosis). The treating specialist variable was divided into six categories; the first five categories included the five most common orthopaedic surgeons working in PC+, and the sixth category included all other orthopaedic surgeons working in PC+. Patient diagnoses were registered by the orthopaedic surgeon after the last consultation in PC+ according to the International Classification of Diseases (ICD-10) [31]. This variable was divided into 12 categories; the first 10 categories included the 10 most common diagnoses in PC+, an 11^th^ category included all other diagnoses and a 12^th^ category included all patients with a missing diagnosis. In addition, the number of MRIs and ultrasounds requested during P2 were described.

### Statistical analysis

To examine the effect of the possibility to request diagnostic tests in PC+, referral decisions and consultation- and patient-related factors were compared between P1 and P2. Continuous data were presented as the mean and 95% confidence interval (CI) and were compared using an independent-sample *t*-test. Categorical data were presented as counts and percentages, and were compared using Pearson’s χ^2^ test, with presenting the 95%CI for 2x2 tables. *P*-values ≤ 0.05 were considered statistically significant.

To describe variations in the referral decision, logistic regression modelling was used, with the decision to refer to outpatient hospital care being a binary yes/no variable. Firstly, univariate logistic regression analysis was used to evaluate the relationship between the referral decision following PC+ and the consultation- and patient-related predictors. Predictors with a *p*-value ≤0.25 were simultaneously entered into the multivariable model (enter method; [32]). Among the categorical predictors, treating specialist and patient diagnosis—the categories with the smallest difference in referral decision between P1 and P2—were selected as the reference group.

The effect of time (P2 vs. P1) on the difference in referral decisions between specialists as well as on the difference in referral decisions between diagnoses were analysed using specialist-period and diagnosis-period interactions, respectively. A significant interaction indicates that the effect of the treating specialist or the patient diagnosis on the referral decisions depends on the period. In this case, interpreting the effect of the individual predictors in isolation can be misleading [33]. The results of the logistic regression were presented as odds ratios (OR) with a 95%CI. *P*-values ≤0.05 were considered statistically significant. The explained variation in the regression model was measured by the Nagelkerke pseudo-R^2^ [34]. All analyses were performed using the SPSS software for Windows, version 25.0 (SPSS Inc., Chicago, IL, USA).

Results of the analyses were discussed during an expert meeting with three involved orthopaedic surgeons. The purpose of this meeting was to verify the findings and to contribute to a better interpretation of the results.

## Results

During the total study period, from January 2015 to December 2017, 2,534 patients visited PC+ for orthopaedic care. The referral decision following PC+ for 96 patients was unknown, so these patients were excluded from the analysis. The remaining 2,438 patients had, in total, 2,766 consultations in PC+, with a mean of 1.13 (95%CI 1.12, 1.14) (Table 2). Following PC+, 67.2% (N = 1,638) of patients were referred back to their GP and 32.8% (N = 800) to outpatient hospital care for further treatment/examination.

**Table 2:** Overview and comparison of PC+ patients and consultation characteristics during P1 and during P2.

	Total (N = 2,438)	P1 (N = 1,384)	P2 (N = 1,054)	Difference between P1 and P2	
				95% CI ^A^	*p*-values
**Referral decision** % (N)				0.40, 0.56	≤ 0.001*
Referral back to GP	67.2 (1,638)	60.3 (834)	76.3 (804)		
Referral to hospital care	32.8 (800)	39.7 (550)	23.7 (250)		
**Age in years** mean (95%CI ^**A**^)	53.4 (52.7, 54.1)	52.7 (51.8, 53.6)	54.4 (53.4, 55.4)	-3.07, -0.35	0.014*
**Gender** % (N)				0.95, 1.31	0.202
Male	43.3 (1,056)	42.2 (584)	44.8 (472)		
Female	56.7 (1,382)	57.8 (800)	55.2 (582)		
**Number of consultations** mean (95%CI ^**A**^)	1.13 (1.12, 1.14)	1.09 (1.07, 1.11)	1.19 (1.17, 1.21)	-0.13, -0.08	≤ 0.001*
**Treating specialist** % (N)				-	≤ 0.001*
Specialist 1	13.0 (318)	14.7 (204)	10.8 (114)		
Specialist 2	11.5 (281)	20.3 (281)	0.0 (0) ^B^		
Specialist 3	10.4 (253)	10.2 (141)	10.6 (112)		
Specialist 4	8.1 (198)	10.0 (139)	5.6 (59)		
Specialist 5	5.3(128)	1.8 (25)	9.8 (103)		
Other specialist	51.7 (1,260)	43.0 (594)	63.6 (666)		
**Patient diagnosis** % (N)				-	≤ 0.001*
Knee osteoarthritis	14.1 (344)	11.8 (164)	17.1 (180)		
Meniscus lesion	7.0 (171)	8.2 (113)	5.5 (58)		
Supraspinatus tendinopathy	6.8 (167)	6.6 (91)	7.2 (76)		
Other enthesopathies	6.2 (150)	6.8 (94)	5.3 (56)		
Pelvis/hip/upper leg osteoarthritis	4.5 (110)	4.6 (64)	4.4 (46)		
Patellofemoral pain syndrome (PFPS)	4.0 (97)	3.8 (53)	4.2 (44)		
Hand/wrist tenosynovitis	3.3 (80)	3.1 (43)	3.5 (37)		
Rotator cuff tears/biceps tendon rupture	2.6 (63)	2.6 (36)	2.6 (27)		
Hand/wrist osteoarthritis	2.6 (63)	2.5 (34)	2.8 (29)		
Spinal osteoarthritis	2.3 (56)	3.5 (48)	0.8 (8)		
Unknown diagnosis	10.8 (264)	9.0 (124)	13.3 (140)		
Other diagnosis	35.8 (873)	37.6 (520)	33.5 (353)		

* p ≤ 0.05

^A^ CI = Confidence Interval

^B^ Specialist did not work at PC+ during this period

During P1, 1,384 patients visited PC+ for orthopaedic care. In total, these patients had 1,507 consultations in PC+, with a mean of 1.09 (95%CI 1.07, 1.11). Following PC+, 60.3% (N = 834) of patients were referred back to their GP and 39.7% (N = 550) to outpatient hospital care for further treatment/examination.

During P2, 1,054 patients visited PC+ for orthopaedic care. In total, these patients had 1,259 consultations in PC+, with a mean of 1.19 (95%CI 1.17, 1.21). Following PC+, 76.3% (N = 804) of patients were referred back to their GP and 23.7% (N = 250) to outpatient hospital care for further treatment/examination.

When comparing both periods, significantly less patients were referred to outpatient hospital care during P2 (95%CI 0.40, 0.56) and patients had significantly more consultations in PC+ during this period (95%CI -0.13, -0.08). Finally, with respect to the treating specialist and patient diagnosis, there was a significant difference in the distribution between P1 and P2 (*p* ≤ 0.001).

### Diagnostic tests

During P2, specialists working in PC+ requested 174 MRIs and 57 ultrasounds. In total, 21.8% (N = 230) of all PC+ patients were referred for an additional diagnostic test.

### Predictors of referral to outpatient hospital care

**Fig 2:**
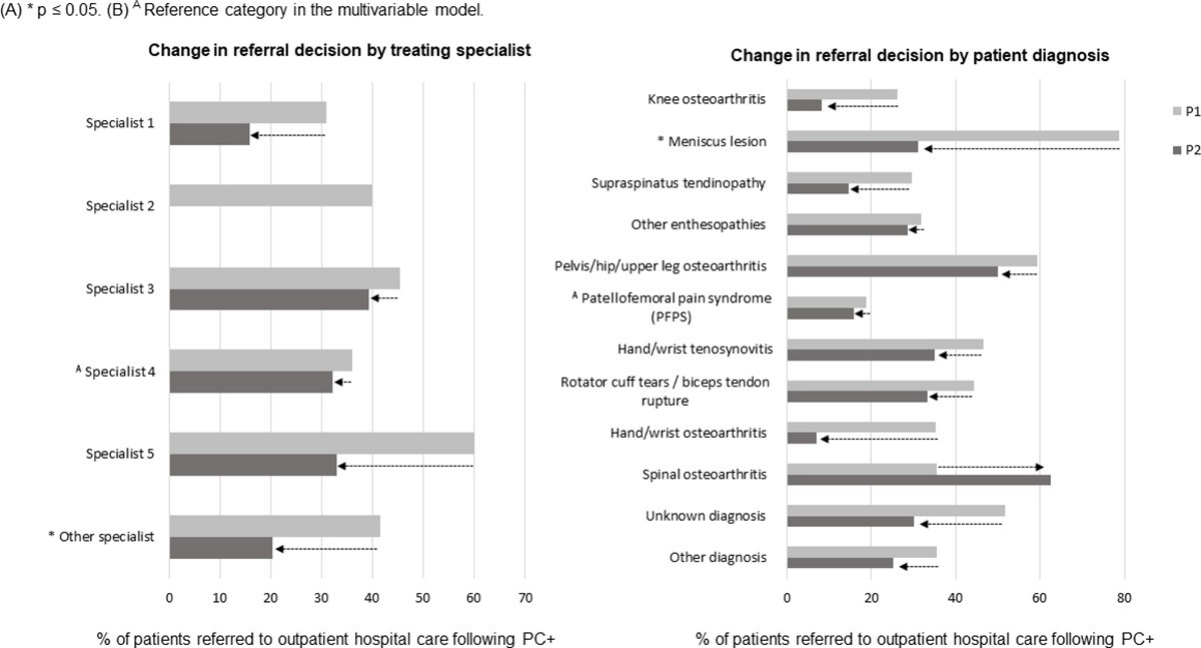
Change in referral decision following PC+ (P1 versus P2).

Specialist 4 and a diagnosis of patellofemoral pain syndrome (PFPS) were selected as the reference group for the logistic regression analysis, because they showed the least change in referral decision following PC+ when P1 and P2 were compared (Fig 2).

Univariate logistic regression analysis was performed to select potential predictors for the multivariable model. Based on the pre-set *p*-value criteria cut-off point of ≤ 0.25, all predictors, with the exception of gender and number of consultations, were included in the multivariable model (Table 3).

**Table 3:** Logistic regression analysis for referral to outpatient hospital care among orthopaedic patients in PC+ (N = 2,438).

	Univariate model		Multivariable model with interaction terms	
	OR (95% CI)	*p*-value	OR (95% CI)	*p*-value
**Age** ^A^	0.86 (0.82, 0.91)	≤.001*	0.86 (0.81, 0.91)	≤0.001**
**Gender(male)**	1.00 (0.85, 1.19)	0.966	…^C^	
**Number of consultations**	0.89 (0.69, 1.14)	0.343	…^C^	
**Period (P2)**	0.47 (0.40, 0.56)	≤.001*	1.72 (0.48, 6.21)	0.409
**Treating Specialist**				
Specialist 1	0.64 (0.43, 0.94)	0.023*	0.87 (0.53, 1.41)	0.561
Specialist 2	1.39 (0.95, 2.05)	0.091*	1.32 (0.77, 2.24)	0.312
Specialist 3	1.24 (0.85, 1.81)	0.266	1.34 (0.85, 2.11)	0.206
Specialist 4	…^B^		…^B^	
Specialist 5	1.16 (0.73, 1.84)	0.529	2.76 (1.08, 7.05)	0.034**
Other specialist	0.81 (0.59, 1.11)	0.192*	1.42 (0.94, 2.15)	0.098
**Patient diagnosis**				
Knee osteoarthritis	0.95 (0.53, 1.73)	0.878	2.34 (1.06, 5.20)	0.037**
Meniscus lesion	7.87 (4.28, 14.45)	≤.001*	19.43 (8.42, 44.83)	≤0.001**
Supraspinatus tendinopathy	1.39 (0.73, 2.62)	0.314	2.51 (1.08, 5.82)	0.032**
Other enthesopathies	2.08 (1.11, 3.90)	0.022*	2.62 (1.14, 6.01)	0.023**
Pelvis/hip/upper leg osteoarthritis	5.86 (3.08, 11.16)	≤.001*	10.05 (4.17, 24.25)	≤0.001**
Patellofemoral pain syndrome (PFPS)	…^B^		…^B^	
Hand/wrist tenosynovitis	3.30 (1.66, 6.57)	0.001*	5.63 (2.21, 14.33)	≤0.001**
Rotator cuff tears/biceps tendon rupture	3.10 (1.50, 6.41)	0.002*	5.67 (2.13, 15.10)	0.001**
Hand/wrist osteoarthritis	1.35 (0.61, 2.97)	0.464	3.25 (1.19, 8.90)	0.022**
Spinal osteoarthritis	3.05 (1.44, 6.44)	0.004*	3.30 (1.30, 8.37)	0.012**
Unknown diagnosis	3.16 (1.77, 5.63)	≤.001*	6.02 (2.68, 13.52)	≤0.001**
Other diagnosis	2.14 (1.24, 3.68)	0.006*	2.91 (1.41, 6.00)	0.004**
**Interaction specialist x period**				0.015**
Specialist 1	-	-	0.43 (0.17, 1.09)	0.076
Specialist 2	-	-	-	-
Specialist 3	-	-	1.05 (0.41, 2.71)	0.924
Specialist 4	-	-	…^B^	
Specialist 5	-	-	0.39 (0.12, 1.28)	0.120
Other specialist	-	-	0.40 (0.19, 0.84)	0.015**
**Interaction diagnosis x period**				≤0.001**
Knee osteoarthritis	-	-	0.29 (0.08, 1.01)	0.052
Meniscus lesion	-	-	0.12 (0.03, 0.45)	0.002**
Supraspinatus tendinopathy	-	-	0.46 (0.12, 1.75)	0.253
Other enthesopathies	-	-	1.10 (0.30, 4.04)	0.890
Pelvis/hip/upper leg osteoarthritis	-	-	0.66 (0.18, 2.50)	0.541
Patellofemoral pain syndrome (PFPS)	-	-	…^B^	
Hand/wrist tenosynovitis	-	-	0.60 (0.15, 2.47)	0.479
Rotator cuff tears/biceps tendon rupture	-	-	0.70 (0.15, 3.14)	0.637
Hand/wrist osteoarthritis	-	-	0.18 (0.03, 1.24)	0.081
Spinal osteoarthritis	-	-	4.86 (0.72, 32.73)	0.104
Unknown diagnosis	-	-	0.33 (0.10, 1.17)	0.086
Other diagnosis	-	-	0.74 (0.24, 2.26)	0.597

OR = the odds ratio; Cl = confidence interval

* p < 0.25 (univariate analysis)

** p < 0.05 (multivariable analysis)

^A^ Age was rescaled such that one unit is equal to 10 years

^B^ Reference category in the multivariable model

^C^ Variable not included in the multivariable model

With respect to the multivariable model, it appeared that with increasing age, the likelihood of patients being referred to outpatient hospital care decreased significantly with OR = 0.86 (95%CI 0.81, 0.91) for every 10 years. Moreover, the multivariable model was adjusted for the possible confounding effect of the difference in age between patients in P1 and P2 (as described in Table 2).

The multivariable model, with the interaction terms included, showed a significant effect for the interaction between the treating specialist and period (*p* = 0.015) and between patient diagnosis and period (*p* ≤ 0.001; Table 3). Regarding the interactions between the treating specialist and the period, the period appeared to have a significantly different effect on the referral behavior to outpatient hospital care for specialists from the category “other specialist” (OR 0.40; 95%CI 0.19,0.84) compared with the effect of the period on the referral behavior of the reference group. As can be seen in Fig 2, Specialists from the category “other specialsit” showed a decrease in the number of referrals to oupatient hosptial care following PC+. Specialists 1 and 5 also showed a strong decrease in the number of referrals to outpatient hospital care (Fig 2), but this decrease was not significant compared with the reference group, which can be explained by the limited number of patients within those categories. The interactions between patient diagnosis and period showed a significantly different effect of the period on patients diagnosed with a meniscus lesion (OR 0.12; 95%CI 0.03, 0.45) compared with the effect of the period on the reference group. As can be seen in Fig 2, this diagnosis showed a strong decrease in the number of patients referred to outpatient hospital care following PC+.

Because of the significant interaction terms, it is not relevant to interpret the isolated effects of period, treating specialist and patient diagnosis. The multivariable model with interaction terms explained 17.6% of the variation (Nagelkerke *R*^*2*^ = 0.176).

## Discussion

The present study evaluated referral decisions following orthopaedic care in PC+, taking into account the influence of the availability of diagnostic tests on the referral decision, as well as consultation- and patient-related predictors of these decisions.

The apparent influence of the possibility to request additional diagnostic tests on the referral decision is in accordance with the study by van Hoof et al. [28]. In the present study, specialists indicated that they needed diagnostic imaging, such as an X-ray, to diagnose patients. During P1 in the current setting of PC+, orthopaedic surgeons required a recent X-ray for all patients prior to the first consultation. Orthopaedic surgeons mentioned that, based on previous experience, approximately 70% of patients would have to obtain an X-ray following the first consultation, so not mandating an X-ray beforehand would make PC+ less effective. Referral rates to outpatient hospital care during this period were, according to the involved stakeholders, still considerable due to the need for additional diagnostic tests. To increase the effectiveness of orthopaedic care in PC+, stakeholders decided to introduce the possibility of requesting additional diagnostic tests, such as MRIs and ultrasounds. As a result, the number of referrals to outpatient hospital care decreased significantly during P2. Despite these positive results, it is important to be wary of unnecessary care in PC+. Because the initial aim of PC+ was to limit the availability of diagnostic tools to promote the generalist approach, it is necessary to determine to what extent diagnostic tests fit within this vision. This is also relevant for ensuring cost-effectiveness and patient-centered care. The number of consultations in PC+ also increased significantly, which can be explained by the fact that specialists needed an extra consultation to discuss the test results with the patient.

Older patients were less likely to be referred to outpatient hospital care following PC+. Similar findings were also reported by McBride et al. [35] in a study on referral variation from primary to secondary care of patients with, among other ailments, hip pain. Possible explanations given in this study were patient preferences and the clinical uncertainty regarding the benefits and adverse effects of treatment for elderly patients [35–37]. Although older patients are slightly more at risk following hip or knee surgery, for example, the quality of life can also increase in this group [38, 39]. In addition, Becker et al. [40] found that younger patients with hand osteoarthritis had a greater likelihood of surgery and also had increased healthcare-related costs.

All specialists showed a decrease in the number of referrals to outpatient hospital care during P2. From the perspective of substituting primary care for specialised medical care, this was the desired effect; however, the extent to which it is desirable to change the referral behavior of specialists when more diagnostic tests are available is questionable. Based on the existing literature and the previous discussion of the extent to which diagnostic tests fit within the vision of PC+, it is important to remain critical towards the availability of diagnostic tests to ensure overuse is not encouraged [41, 42]. According to Vierhout et al. [24], diagnostic tests carried out by the orthopaedic suregeon are not always needed and they might be requested based on routine. The availability of diagnostic tests in PC+ should therefore be a topic for discussion among the involved stakeholders, and the awareness among specialists regarding the necessity of diagnostic tests should be enhanced [43].

The novelty of the PC+ setting also requires specialists to deal with a new context and environment as well as their related expectations and understandings of what best practice is in this setting [44]. PC+ involves more than shifting outpatient hospital care to the primary setting; it is also about changing the mind-sets and behaviour of the involved health care professionals—both the GPs and medical specialists [45]. As described by Gupta et al. [44], changing clinical practice is a complex process of learning and unlearning. The degree to which medical specialists succeed in changing their behavior according to the PC+ setting is questionable. Based on our findings about PC+, practice patterns varied among the involved orthopaedic surgeons. More research is therefore needed to specify relevant features of medical specialists to work in PC+, taking into account the specific setting of PC+. The number of medical specialists working in PC+ is a related area for discussion. In total, 37 different specialists (orthopaedic surgeons and specialty trainees) worked in PC+ during the study period. This number indicates a high turnover of specialists, which could be a barrier to the development of practice patterns in this new setting and could limit any possible learning effect between specialists and GPs or the opportunity to overcome the knowledge gap in primary care [19]. Stimulating collaboration between medical specialists and GPs may be associated with improved health outcomes, optimised care, and less use of hospital care [46], so assigning a select group of appropriate specialists for PC+ could improve the effectivenes of the program.

Regarding patient diagnosis, several diagnoses showed a decrease in referrals to secondary care during P2, but patients diagnosed with spinal osteoarthiritis showed an increase in referrals to secondary care during P2, which indicates that spinal osteoarthritis appears to be less appropriate for PC+ even when additional diagnostic tests are available. During the expert meeting, specialists confirmed this assumption. Patients with back problems should not be referred to PC+ for orthopaedic care, but should be referred to a specific back pain clinic. Development of patient profiles for PC+ appears relevant for further optimisation of the program. These profiles can give an indication of patient complaints that are suitable for PC+, which would support GPs in their PC+ referral behavior [28].

### Limitations

The variation of 17.6% explained by the final model in the present study suggests that many factors influence referral decisions following PC+. The number of predictors included in the present study was restricted, which was inherent to the use of monitoring data containing a limited amount of information. More information, such as the International Classification of Primary Care (ICPC) codes [47] and registration of the severity of the complaint, would likely lead to a better prediction of the referral decision and therefore be more recognisable and manageable for GPs. Accordingly, appropriate referrals to PC+ will increase, and consequentially so will the intervention’s efficiency.

Moreover, a large number of different diagnoses were determined by the specialists in PC+ (N = 138). Only the 10 most common diagnoses were divided in separate categories, accounting for 53% of all consultations. The remaining diagnoses (N = 128) were merged into an 11^th^ category, accounting for 36% of all consultations. Additionally, the diagnoses of 264 patients (11%) were missing, which was partly caused by the specialists becoming accustomed to the registration method at the beginning of PC+, but this is not supposed to influence the results considerably.

## Conclusions

The results of this study reveal that the possibility of requesting additional diagnostic tests for orthopaedic surgeons working in a primary care setting significantly decreased the number of referrals to outpatient hospital care. With more than three-quarter of the patients referred back to their GP during P2, orthopaedic care seems to fit to the aim of PC+ to prevent unnecessary referrals to hospital care. However, more research is needed regarding the effectiveness and suitability of the use of diagnostic tests to further optimise orthopaedic care in PC+. Selection of the appropriate profiles to indicate suitable specialists and patients for PC+ is therefore recommended, because both significantly influenced the referral decision. Other factors such as volume, planning and duration of consultations, quality of care, patient health status, and cost of care should also be taken into account in future research to further optimise the substitution of orthopaedic care and reduce rising healthcare costs.
